# Complete genome sequence of *Leptotrichia buccalis* type strain (C-1013-b^T^)

**DOI:** 10.4056/sigs.1854

**Published:** 2009-09-24

**Authors:** Natalia Ivanova, Sabine Gronow, Alla Lapidus, Alex Copeland, Tijana Glavina Del Rio, Matt Nolan, Susan Lucas, Feng Chen, Hope Tice, Jan-Fang Cheng, Elizabeth Saunders, David Bruce, Lynne Goodwin, Thomas Brettin, John C. Detter, Cliff Han, Sam Pitluck, Natalia Mikhailova, Amrita Pati, Konstantinos Mavrommatis, Amy Chen, Krishna Palaniappan, Miriam Land, Loren Hauser, Yun-Juan Chang, Cynthia D. Jeffries, Patrick Chain, Christine Rohde, Markus Göker, Jim Bristow, Jonathan A. Eisen, Victor Markowitz, Philip Hugenholtz, Nikos C. Kyrpides, Hans-Peter Klenk

**Affiliations:** 1 DOE Joint Genome Institute, Walnut Creek, California, USA; 2 DSMZ - German Collection of Microorganisms and Cell Cultures GmbH, Braunschweig, Germany; 3 Los Alamos National Laboratory, Bioscience Division, Los Alamos, New Mexico USA; 4 Biological Data Management and Technology Center, Lawrence Berkeley National Laboratory, Berkeley, California, USA; 5 Oak Ridge National Laboratory, Oak Ridge, Tennessee, USA; 6 Lawrence Livermore National Laboratory, Livermore, California, USA; 7 University of California Davis Genome Center, Davis, California, USA

**Keywords:** *Fusobacteria*, '*Leptotrichiaceae*', Gram-negative fusiform rods, human oral microflora, dental plaque, non-motile, non-sporulating, anaerobic

## Abstract

*Leptotrichia buccalis* (Robin 1853) Trevisan 1879 is the type species of the genus, and is of phylogenetic interest because of its isolated location in the sparsely populated and neither taxonomically nor genomically adequately accessed family '*Leptotrichiaceae*' within the phylum '*Fusobacteria*'. Species of *Leptotrichia* are large, fusiform, non-motile, non-sporulating rods, which often populate the human oral flora. *L. buccalis* is anaerobic to aerotolerant, and saccharolytic. Here we describe the features of this organism, together with the complete genome sequence and annotation. This is the first complete genome sequence of the order '*Fusobacteriales*' and no more than the second sequence from the phylum '*Fusobacteria*'. The 2,465,610 bp long single replicon genome with its 2306 protein-coding and 61 RNA genes is a part of the *** G****enomic* *** E****ncyclopedia of* *** B****acteria and* *** A****rchaea * project.

## Introduction

Strain C-1013-b^T^ (= DSM 1135 = ATCC 14201 = JCM 12969) is the type strain of *Leptotrichia buccalis* [1], which is the type species of the genus first adequately described in 1879 by Trevisan to accommodate the oral filamentous bacteria and to separate them from the algae [2,3]. For a while, two entirely different organisms were termed *L. buccalis* in the literature [3]. One of these was '*Leptothrix buccalis'*, a name originally employed by Robin in 1853 for filamentous forms which he had seen in wet mounts of tooth scrapings [4]. Over a century of the history of classification and misclassification of *L. buccalis* was documented by Gilmore *et al.* 1961 [3]. *L. buccalis* was among the first bacteria to be described and drawn in the letters of Antoni van Leeuwenhoek [5]. Next to *Fusobacterium nucleatum* [6], *L. buccalis* is only the second species from the phylum *Fusobacteria* for which a complete genome sequence is described. Here we present a summary classification and a set of features for *L. buccalis* strain C-1013-b^T^ together with the description of the complete genomic sequencing and annotation.

## Classification and features

**Figure 1:**
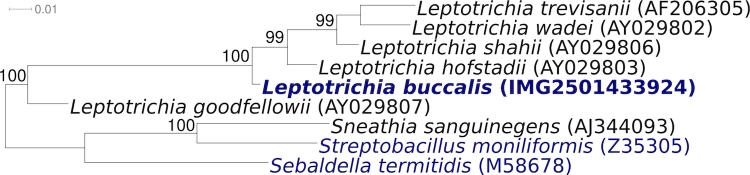
Phylogenetic tree highlighting the position of *L. buccalis* C-1013-b^T^ relative to all type strains of the genus *Leptotrichia* inferred from 1,421 aligned characters [15,16] of the 16S rRNA sequence under the maximum likelihood criterion [17], and rooted with all type strains of the family *Leptotrichiaceae*. The branches are scaled in terms of the expected number of substitutions per site. Numbers above branches are support values from 1,000 bootstrap replicates, if larger than 60%. Lineages with type strain genome sequencing projects registered in GOLD [18] are shown in blue, published genomes in bold.

The primary habitat of *L. buccalis* and most other *Leptotrichia* species is the human oral cavity, especially dental plaque. *L. buccalis* also is found in the female genitourinary tract and the intestinal tract [11,13]. Although *L. buccalis* and *L. buccalis*-like bacteria have also occasionally been recovered from blood, mostly in immunocompromised patients, they are not known as causative agents of systemic infections [11,14] even though an endotoxin was documented for the *L. buccalis* [5,14]. Almost all of the cultivated *Leptotrichia* isolates cluster in 16S rRNA sequence comparisons with one of the five other type strains of the genus *Leptotrichia* [11] (Figure 1). Except for the uncultured clone GI5-008-C04 (FJ192568), which has been recovered from screening of a spacecraft assembly clean room during the Phoenix mission, all significantly related phylotypes were from the usual habitats as described above. No phylotypes from environmental screening or genomic surveys could be linked with more than 85% 16S rRNA sequence similarity to *L. buccalis* (status May 2009).

Figure. 1 shows the phylogenetic neighborhood of *L. buccalis* strain C-1013-b^T^ in a 16S rRNA based tree. The sequences of the five 16S rRNA gene copies in the genome of strain C-1013-b^T^ differ from each other by 5 to 20 nucleotides (up to 1.3%), and by 4 to 16 nucleotides plus 38 ambiguities (total up to 3.6%) from the previously published 16S rRNA sequence generated from NCTC 10429 (X90831).

**Figure 2:**
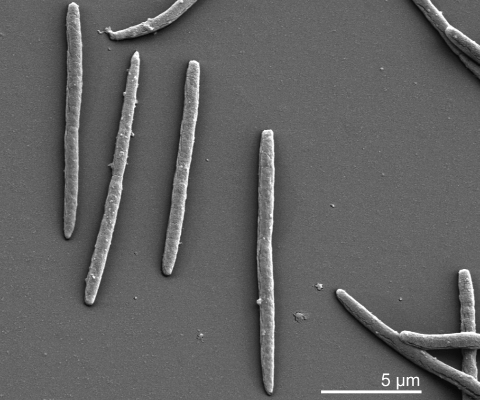
Scanning electron micrograph of *L. buccalis* stain C-1013-b^T^ (Manfred Rohde, Helmholz Centre for Infection Research, Braunschweig)

Older cells of *L. buccalis* strain C-1013-b^T^ are Gram negative, but younger cells that have been in culture for less than six hours are Gram-positive Table 1 [5]. The organism forms long rods, commonly occurring in pairs, and is non-motile [5] (Figure 2). Young colonies are colorless, smooth, shiny, raised and described as “medusa-head” colonies because of filamentous edges [5]. On first isolation, *L. buccalis* is anaerobic but becomes aerotolerant upon transfer and grows in the presence of air and CO_2_ [5,13]. *L. buccalis* is susceptible to many antibiotics but resistant to aminoglycosides [5]. The organism is highly saccharolytic and ferments a range of different sugars [5,13]. The main metabolic end product is lactic acid [13]. The G+C content was already described in 1982 as ‘unusually low’ (25%) [5].

**Table 1:** Classification and general features of *L. buccalis* strain C-1013-b^T^ according to the MIGS recommendations [7]

**MIGS ID**	**Property**	**Term**	**Evidence code**	
	Current classification	Domain *Bacteria*	TAS [8]	
		Phylum *'Fusobacteria'*	TAS [9]	
		Class *'Fusobacteria'*	TAS [9]	
		Order *'Fusobacteriales'*	TAS [9]	
		Family *'Leptotrichiaceae'*		
		Genus *Leptotrichia*	TAS [2]	
		Species *Leptotrichia buccalis*	TAS [2]	
		Type strain C-1013-b	TAS [1]	
	Gram stain	negative	TAS [5]	
	Cell shape	long rods	TAS [5]	
	Motility	nonmotile	TAS [5]	
	Sporulation	nonsporulating	TAS [5]	
	Temperature range	mesophile	NAS	
	Optimum temperature	37°C	NAS	
	Salinity	normal	NAS	
MIGS-22	Oxygen requirement	anaerobic on isolation, becomes aerotolerant on further transfer	TAS [5]	
	Carbon source	mono- and disaccharides	TAS [5]	
	Energy source	carbohydrates	NAS	
MIGS-6	Habitat	oral cavities	TAS [5]	
MIGS-15	Biotic relationship	free living	NAS	
MIGS-14	Pathogenicity	opportunistic pathogen	TAS [5]	
	Biosafety level	1	TAS [10]	
	Isolation	human oral flora	TAS [11]	
MIGS-4	Geographic location	global	NAS	
MIGS-5	Sample collection time	mid of 19^th^ century	TAS [3]	
MIGS-4.1 MIGS-4.2	Latitude – Longitude	not reported		
MIGS-4.3	Depth	not reported		
MIGS-4.4	Altitude	not reported		

Evidence codes - IDA: Inferred from Direct Assay (first time in publication); TAS: Traceable Author Statement (i.e., a direct report exists in the literature); NAS: Non-traceable Author Statement (i.e., not directly observed for the living, isolated sample, but based on a generally accepted property for the species, or anecdotal evidence). These evidence codes are from the Gene Ontology project [12]. If the evidence code is IDA, then the property was observed for a living isolate by one of the authors or an expert mentioned in the acknowledgements.

### Chemotaxonomy

The murein of strain C-1013-b^T^ contains *meso*-2,6-diaminopimelic acid (A_2_pm), D- and L-alanine, and D-glutamic acid [19]. The strain possesses muramic acid and glucosamine as principal components of its peptidoglycan [19]; type A1γ according to the classification of Schleifer and Kandler [20]. As in other *Leptotrichia* strains, the fatty acid pattern of *L. buccalis* is an almost equal mixture of saturated and unsaturated straight chain acids: C_16:0_ (39%), C_14:0_ (10%), C_18:1_ (42%), with about 7% hydroxy acids (C_14:0_) [11]. The type of menaquinones and polar lipids used by *L. buccalis* has not been described yet.

## Genome sequencing

### Genome project history

This organism was selected for sequencing on the basis of its phylogenetic position, and is part of the *** G****enomic* *** E****ncyclopedia of* *** B****acteria and* *** A****rchaea * project. The genome project is deposited in the Genomes OnLine Database [18] and the complete genome sequence is deposited in GenBank. Sequencing, finishing and annotation were performed by the DOE Joint Genome Institute (JGI). A summary of the project information is shown in Table 2.

**Table 2:** Genome sequencing project information

**MIGS ID**	**Property**	**Term**
MIGS-31	Finishing quality	Finished
MIGS-28	Libraries used	Three genomic libraries: two Sanger libraries - 8 kb pMCL200 and fosmid pcc1Fos and one 454 pyrosequence standard library
MIGS-29	Sequencing platforms	ABI3730, 454 GS FLX
MIGS-31.2	Sequencing coverage	9.7× Sanger; 42× pyrosequence
MIGS-30	Assemblers	Newbler version 1.1.02.15, phrap
MIGS-32	Gene calling method	Prodigal
	INSDC / Genbank ID	CP001685
	Genbank Date of Release	September 1, 2009
	GOLD ID	Gc01090
	NCBI project ID	29445
	Database: IMG-GEBA	2501416906
MIGS-13	Source material identifier	DSM 1135
	Project relevance	Tree of Life, GEBA

### Growth conditions and DNA isolation

*L. buccalis* strain C-1013-b^T^ (DSM 1135) was grown anaerobically in DSMZ medium 104 (modified PYG-Medium, http://www.dsmz.de/microorganisms/medium/pdf/DSMZ_Medium104.pdf) at 37°C. DNA was isolated from 1-1.5 g of cell paste using Qiagen Genomic 500 DNA Kit (Qiagen, Hilden, Germany) following the manufacturer's instructions

### Genome sequencing and assembly

The genome was sequenced using a combination of Sanger and 454 sequencing platforms. All general aspects of library construction and sequencing performed at the JGI can be found at http://www.jgi.doe.gov/. 454 Pyrosequencing reads were assembled using the Newbler assembler version 1.1.02.15 (Roche). Large Newbler contigs were broken into 2,747 overlapping fragments of 1,000 bp and entered into assembly as pseudo-reads. The sequences were assigned quality scores based on Newbler consensus q-scores with modifications to account for overlap redundancy and to adjust inflated q-scores. A hybrid 454/Sanger assembly was made using the parallel phrap assembler (High Performance Software, LLC). Possible mis-assemblies were corrected with Dupfinisher or transposon bombing of bridging clones [21]. Gaps between contigs were closed by editing in Consed, custom primer walk or PCR amplification. A total of 908 Sanger finishing reads were produced to close gaps, to resolve repetitive regions, and to raise the quality of the finished sequence. The error rate of the completed genome sequence is less than 1 in 100,000. Together all sequence types provided 51.7× coverage of the genome. The final assembly contains 28,754 Sanger reads in addition to the 454 based pseudo reads.

### Genome annotation

Genes were identified using Prodigal [22] as part of the Oak Ridge National Laboratory genome annotation pipeline, followed by a round of manual curation using the JGI GenePRIMP pipeline (http://geneprimp.jgi-psf.org) [23]. The predicted CDSs were translated and used to search the National Center for Biotechnology Information (NCBI) nonredundant database, UniProt, TIGRFam, Pfam, PRIAM, KEGG, COG, and InterPro databases. Additional gene prediction analysis and functional annotation was performed within the Integrated Microbial Genomes (IMG-ER) platform [24].

### Genome properties

**Figure 3:**
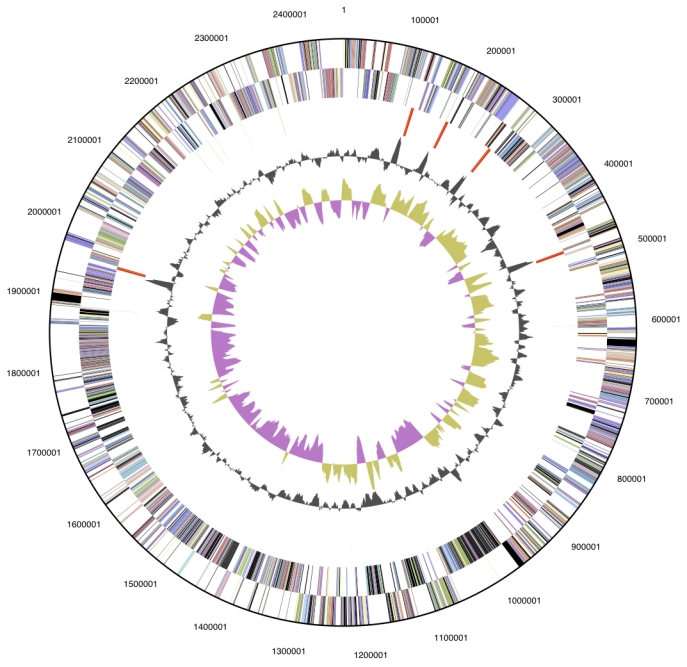
Graphical circular map of the genome. From outside to the center: Genes on forward strand (color by COG categories), Genes on reverse strand (color by COG categories), RNA genes (tRNAs green, rRNAs red, other RNAs black), GC content, GC skew.

The genome is 2,345,610 bp long and comprises one circular chromosome with a 29.7% GC content (Table 3 and Figure 3). Of the 2367 genes predicted, 2306 were protein coding genes, and 61 RNAs; 86 pseudogenes were also identified. The majority of the genes (65.4%) were assigned with a putative function while those remaining were annotated as hypothetical proteins. The properties and the statistics of the genome are summarized in Table 3. The distribution of genes into COGs functional categories is presented in Table 4.

**Table 3:** Genome Statistics

**Attribute**	Value	% of Total
Genome size (bp)	2,465,610	100.00%
DNA Coding region (bp)	2,139,206	86.76%
DNA G+C content (bp)	730,947	29.65%
Number of replicons	1	
Extrachromosomal elements	0	
Total genes	2,367	100.00%
RNA genes	61	2.58%
rRNA operons	5	
Protein-coding genes	2,306	97.42%
Pseudo genes	86	3.63%
Genes with function prediction	1,547	65.36%
Genes in paralog clusters	402	16.98%
Genes assigned to COGs	1,,533	64.77%
Genes assigned Pfam domains	1,577	66.62%
Genes with signal peptides	432	18.25%
Genes with transmembrane helices	530	22.39%
CRISPR repeats	4	

**Table 4:** Number of genes associated with the general COG functional categories

Code	Value	% of total	Description
J	144	6.2	Translation, ribosomal structure and biogenesis
A	0	0.0	RNA processing and modification
K	86	3.7	Transcription
L	124	5.4	Replication, recombination and repair
B	0	0.0	Chromatin structure and dynamics
D	24	1.0	Cell cycle control, mitosis and meiosis
Y	0	0.0	Nuclear structure
V	28	1.2	Defense mechanisms
T	47	2.0	Signal transduction mechanisms
M	112	4.9	Cell wall/membrane biogenesis
N	6	0.3	Cell motility
Z	0	0.0	Cytoskeleton
W	0	0.0	Extracellular structures
U	34	1.5	Intracellular trafficking and secretion
O	68	3.0	Posttranslational modification, protein turnover, chaperones
C	79	3.4	Energy production and conversion
G	110	4.8	Carbohydrate transport and metabolism
E	176	7.6	Amino acid transport and metabolism
F	54	2.3	Nucleotide transport and metabolism
H	80	3.5	Coenzyme transport and metabolism
I	44	1.9	Lipid transport and metabolism
P	84	3.6	Inorganic ion transport and metabolism
Q	11	0.5	Secondary metabolites biosynthesis, transport and catabolism
R	206	8.9	General function prediction only
S	141	6.1	Function unknown
-	773	28.1	Not in COGs
